# Exploring Young People’s Perceptions of the Effectiveness of Text-Based Online Counseling: Mixed Methods Pilot Study

**DOI:** 10.2196/13152

**Published:** 2019-07-03

**Authors:** Pablo Navarro, Matthew Bambling, Jeanie Sheffield, Sisira Edirippulige

**Affiliations:** 1 School of Psychology The University of Queensland Brisbane Australia; 2 Kids Helpline Yourtown Brisbane Australia; 3 Central School of Medicine Royal Brisbane and Women's Hospital The University of Queensland Brisbane Australia; 4 Centre for Online Health The University of Queensland Brisbane Australia

**Keywords:** mental health, child health, adolescent health, distance counseling, mhealth, applied psychology, psychological processes

## Abstract

**Background:**

Young people aged 10-24 years are at the highest risk for mental health problems and are the least likely to seek professional treatment. Owing to this population’s high consumption of internet content, electronic mental (e-mental) health services have increased globally, with an aim to address barriers to treatment. Many of these services use text-based online counseling (TBOC), which shows promising results in supporting young people but also greater variance in outcomes compared with adult comparators.

**Objective:**

This pilot study qualitatively explored the characteristics of users aged 15-25 years accessing TBOC services, their motivations for access, and their perceptions about factors believed to influence the effectiveness of these modalities.

**Methods:**

E-surveys were administered naturalistically to 100 young service users aged 15-25 years who accessed webchat and email counseling services via an Australian e-mental health service. Thematic analysis of qualitative themes and quantitative descriptive and proportional data presented in electronic surveys were examined across the areas of user characteristics, motivations for selecting TBOC modalities, and their perceptions of TBOC effectiveness.

**Results:**

Participants were predominately female high school students of Caucasian or European descent from middle socioeconomic status, living with their parents in major cities. Four domains and various themes and subthemes were related to participants’ reasons for accessing TBOC and perceptions of its effectiveness: user characteristics (ie, physical and mental health syndrome and perceived social difficulties), selection factors (ie, safety, avoidance motivation, accessibility, and expectation), factors perceived to increase effectiveness (ie, general therapeutic benefits, positive modality and service factors, and persisting with counseling to increase benefit), and factors perceived to decrease effectiveness (ie, negative modality and service factors, and persisting with counseling despite benefit).

**Conclusions:**

Participants were motivated to use TBOC to increase their sense of safety in response to negative perceptions of their social skills and the response of the online counsellor to their presenting problem. By using TBOC services, they also sought to improve their access to mental health services that better met their expectations. Factors that increased effectiveness of TBOC were the counsellor’s interpersonal skills, use of text-based communication, and persisting with beneficial counseling sessions. Factors that reduced TBOC effectiveness were poor timeliness in response to service requests, experiencing no change in their presenting problem, not knowing what postcounseling action to take, and persisting with ineffective counseling sessions.

## Introduction

### Background

Population-based prevalence and cross-cultural studies indicate that young people aged 10-24 years are at the highest risk for developing emotional and mental health problems [1-8] and are also the least likely to seek professional treatment [9-14]. Since many mental health disorders become more serious over time [1,15,16] and can benefit from early treatment [17-19], the underutilization of mental health services by young people has sparked considerable academic interest [20].

Several barriers to mental health help seeking have been identified for young people including knowledge-based barriers related to mental health literacy and service awareness; structural barriers related to service accessibility and affordability; and sociocognitive barriers related to beliefs about the nature of mental ill health, how one should manage problems, and concerns about the help-seeking process [21-26]. Academic interest has therefore turned to internet-based interventions owing to the advantages in their cost-effectiveness and accessibility over conventional service delivery, which is believed to reduce the aforementioned barriers to care [27-29]. Consequently, a network of electronic mental (e-mental) health services has emerged in the international market, seeking to improve the accessibility of services to young people [30,31], many of which have been adopted into national mental health strategies [32-34].

Although face-to-face counseling remains the favored modality for most young people seeking mental health support [10], an increasing number have expressed a preference for text-based online counseling (TBOC) using asynchronous (eg, email, short message service [SMS], and forum) and synchronous (eg, webchat) modalities [35,36]. Literature examining the effectiveness of these modalities in supporting young people is in its relative infancy and shows mixed but generally promising findings [37-41]. In comparison with the adult e-mental health literature, these findings also appear to show relatively greater variance [42-47]. This raises important questions about the aspects of TBOC that may make it less effective for young people, which requires an understanding of the characteristics of young service users, their motivations for access, and their perception of factors that affect counseling outcome.

Despite the paucity of TBOC literature for supporting young people, existing research offers several useful insights. Studies indicate that the demographic background of young service users on TBOC modalities majorly (80%) comprises female-identifying 14- to 17-year-old adolescents from urban locations [40,48-51]. Some research also suggests that a significant proportion of young people using TBOC may be one-off service users [40,50,51] or accessing more than one mental health service [50]. Studies indicate that the motivations underpinning young people’s selection of TBOC modalities center on perceptions of its heightened safety due to environmental privacy, anonymity, autonomy, control, and emotional distance from the counsellor as well as its increased accessibility due to its convenience, ease of access, and affordability [35,38,49,52-55]. Other motivations include a preference for text communication and polarized minimal or heightened expectations about the outcome of counseling [37,56,57]. Studies indicate that most common presenting problems for young people accessing TBOC are related to mental health, relationships, and information-related requests (eg, medico-legal questions, service referrals, and resource requests) [31,38,39,48,58], with some evidence suggesting that service users experience a higher level of distress or perceived burden of the problem than those presenting on telephone-based modalities [39,50,51]. Finally, at least one study found that young people perceived several obstacles to obtaining positive outcomes using TBOC, including online counsellors misunderstanding their content and emotions, difficulty capturing the online counsellor’s mood, challenges building a therapeutic alliance, poor timeliness in responses to service requests, and not having enough time to work on a problem [49].

Several themes are striking with regard to factors that may explain the variance in TBOC effectiveness for young people. First, the user characteristics and motivations of young people suggest that many are early help seekers desiring safety or control over service use, which may indicate issues with readiness and motivation for change. Many young people also appear to have minimal or heightened expectations about counseling outcome while presenting with high distress or complex problems, which may indicate a poor fit between the complexity of presenting problems and what is possible at TBOC services, given their modality-specific limitations. Finally, young people appear to struggle with a number of service-modality factors common to TBOC services, such as building a therapeutic alliance in a text-based environment, poor timeliness in response to their service requests, and not having enough time to work on a problem. With the popularity of e-mental health services for young people across the globe, it is imperative to investigate the factors that increase and decrease the effectiveness of these services.

### Objective

The primary aim of this study was to confirm the following domains and themes indicated by the literature about young service users’ TBOC experiences for measurement in a larger future study:

- User characteristics (eg, age, gender, geographic location, and physical and mental health status)
- Motivations for selecting TBOC (eg, safety, accessibility, and expectation)
- Therapeutic benefits experienced during sessions (eg, catharsis and validation)
- Experiences of ineffectiveness (eg, not making any progress)
- Reasons for persisting with counseling when perceived to be ineffective

The secondary aim of this study was to expand on areas endorsed by participants in order to identify additional constructs of interest for our future study. It was expected that young service users would endorse each of the domains and themes being measured in line with the existing literature. Although similar literature exists, to our knowledge, no study has specifically investigated young people’s perceptions about what decreases the effectiveness of TBOC services. It is believed that investigating effectiveness from this perspective will allow for a more in-depth analysis of what contributes to the variance in effectiveness observed in TBOC outcome literature.

## Methods

### Design

The study utilized a naturalistic mixed methods approach using electronic surveys (e-surveys) to collect data about young service users and their motivations and perceptions of TBOC effectiveness relative to face-to-face services, where applicable. It was reasoned that to naturalistically gather these data, while facilitating the well-documented desire for privacy and anonymity in this population, individual surveying techniques were best suited to this investigation [59].

E-surveys were informed by previous literature and were designed to confirm the aforementioned domains and themes by using four common steps:

- The presentation of information about a target TBOC domain
- Asking participants to rate whether they had ever experienced themes in this domain when utilizing TBOC services on a dichotomous Yes/No scale
- Asking participants that endorsed the target domain or themes to rate its significance to them on a 5-point Likert scale
- Asking participants that endorsed the target domain or themes to elaborate on their experiences, or to identify new related TBOC domain or themes

The full list of survey items can be found in Multimedia Appendix 1.

### Participants

Participants were a naturalistic sample of 359 young Australians accessing *Kids Helpline*, a national e-mental health service in Australia for young people aged 5-25 years. Exclusion criteria in the study were age < 15 years and never having used TBOC at an e-mental health service, which resulted in 148 young people being disqualified from the study. Exclusion criteria were selected to improve the quality of responses by minimizing literacy issues associated with very young age and inexperience with using various TBOC services. A total of 107 participants who had substantial missing data (>50%) and four participants who had missing data related to the core construct measurement had their data removed from the analyses.

### Procedure

Ethical approval for the present study was obtained from the University of Queensland’s Behavioural & Social Sciences Ethical Review Committee. Participants were recruited via an advertisement in the “virtual waiting room” of the webchat counseling service or in the footer of emails exchanged between user and counsellor as part of the email counseling service. Participants were asked to complete an e-survey hosted external to the service (ie, Survey Gizmo). Participant responses were downloaded by researchers directly from the e-survey software.

### Data Analysis

Descriptive statistics and thematic analysis (ie, deductive and inductive) were the primary approaches for understanding patterns and themes in participants’ perceptions about the effectiveness of TBOC. According to Braun and Clarke [60], thematic analysis typically involves the process of becoming familiar with data, generating initial thematic codes, searching for themes, reviewing themes, defining and naming themes, and producing a report. Descriptive statistics that informed deductive themes were calculated within the e-survey software. Qualitative data were analyzed manually by the primary author who identified and defined a set of preliminary themes. A second independent researcher engaged in the same analytic process, and coding discrepancies were discussed until a consensus was obtained. Qualitative themes were only rated once for each participant describing them, irrespective of the number of times that theme was discussed. The final stages of analysis involved the authors reviewing themes and subthemes to ensure that coded extracts were valid, logical, and reasoned.

The magnitude of themes was rated as *very strong*, *strong, moderate,* and *weak* if >80%, >50%, >30%, >10% of participants offering quantitative (N=100) or qualitative (N=70) data endorsed the theme, respectively, with cut-offs drawn from the effect size literature. Quantitative ratings of *very important* and *important* were aggregated. Where frequency data for a theme existed quantitatively and qualitatively, the highest percentage was assigned a magnitude rating and a percentage range. Themes endorsed by fewer than 10% of all participants were excluded from the analysis on the grounds of low generality.

## Results

### Overview

**Figure 1:**
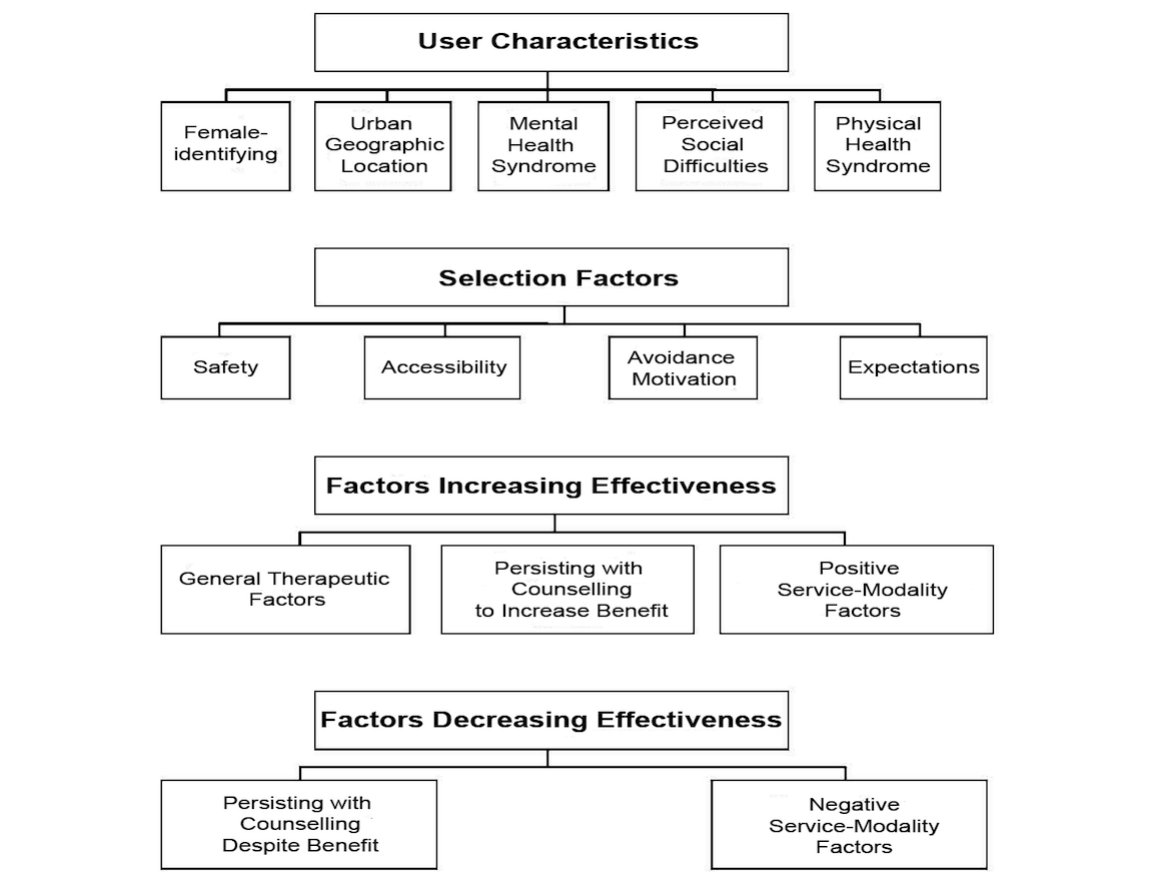
Nonhierarchical overview of primary domains and themes identified in the study.

Four domains were confirmed to be related to participants’ user characteristics, their motivations for selecting TBOC, and their perceptions of its effectiveness: *user characteristics*, *selection factors*, *factors perceived to increase effectiveness*, and *factors perceived to decrease effectiveness*. Figure 1 shows an illustrative overview of primary domains and themes identified in the study.

### User Characteristics

Participants confirmed three themes related to their *user characteristics*: *gender*, *geographic location*, and *mental and physical health status*. A fourth theme, *perceived social difficulties*, emerged from participant qualitative data. An overview of the themes related to the *user characteristics* domain can be found in Table 1.

The final sample consisted of 100 participants aged between 15 and 25 years (mean 19.6, SD 3.2). Of this number, 81 were female, 64 were of Caucasian or European descent, 59 followed no religion, and 76 were not in a committed relationship. Sixty-seven participants identified their primary vocational role as a student, and 71 participants were in high school or had a partial high school education. In addition, 57 participants were living with both parents. Most participants were also accessing the services from major Australian cities, with 28 living in New South Wales, 24 living in Victoria, and 22 living in Queensland. More than half the sample was estimated to be from a middle socioeconomic status based on parental education proxy indicators, where 58 and 59 participants had one or two parents, respectively, who had completed schooling below university level. Eighty-eight participants reported using the noncall and internet features of their devices on a daily or near-daily basis. Seventy participants had used webchat counseling previously, 23 participants had used both webchat and email counseling previously, and 7 participants had only ever used email counseling. Participants also had a mix of experience using webchat or email counseling, with 41 having used these services 2-4 times, 32 having used these services 4-10 times, and 27 having used these services ≥10 times. There were no demographic differences between participants who provided qualitative responses (n=70) and those who did not, with the exception of fewer responses from short- and long-term users.

About less than half of the sample (46/100) reported having been diagnosed with a *mental health syndrome*. The most common mental health syndromes were anxiety (35/100) and mood (30/100) syndromes, followed by personality (12/100), posttraumatic stress (11/100), and eating syndromes (10/100). Twelve participants reported having been diagnosed with a chronic *physical health syndrome*. The most common physical health syndromes were respiratory disease (5/100) and other nonspecified diseases (5/100).

*Having perceived social difficulties* that made it challenging to access nontext counseling modalities emerged as a qualitative theme (17/70, 24% of participants). Participant responses were variable. Key aspects that emerged were anxiety and fear when communicating verbally, a perceived deficit in communication skills, and a lack of confidence with verbal communication.

**Table 1:** Overview of the themes related to the *user characteristics* domain that were confirmed and identified in the study.

Themes	Strength	Examples
Female-identifying gender	Very strong	—^a^
Urban geographic location	Strong	—
Mental health syndrome	Moderate	—
Perceived social difficulties	Moderate	“It's hard for me to open up to people because I don't know how to start the conversation with somebody face to face, especially because I'm an awkward and occasionally shy person”
Physical health syndrome	Weak	—

^a^Not available.

**Table 2:** Overview of themes and subthemes related to the *selection factors* domain that were confirmed and identified in the study.

Themes and subthemes		Strength	Examples
**Safety**		Very strong	
	Increased privacy	Very strong	“Privacy is important to me because I reallyyyy do not want my parents to know... well, anything”, “It’s difficult to have a phone conversation about delicate matters when others are around but no one can over hear a web chat”
	Facilitates honesty or communication of sensitive issues	Very strong	“I can talk about things I would otherwise be too scared to talk about like past sexual abuse”
	Increased anonymity	Strong	“I feel in control of things better being anonymous it's easier to chat about hard things”
	Reducing the emotional intensity of conversation	Strong	“Less confronting, as phone calls I find hard to deal with and I feel less self-conscious on a webchat”
	Increasing control and autonomy	Strong	“I needed to feel like I could control the conversation, like I could say whatever I wanted and not feel judged”
**Avoidance motivation**		Strong	
	Being overheard or seen attending a service	Moderate	“I did not want my family to find out that I needed to talk to someone. as far as they were concerned I was happy”
	Social interaction	Weak	“I don't really like talking to people or people who help so I found the online service very helpful. They could still get an idea of your issue but you didn't have to go back or be face to face”
	Minimize intense Or difficult emotions	Weak	“Because I get teary easily and I don't like crying whilst trying to talk to someone”
	Counsellor reaction	Weak	“It's so much easier to talk o counsellors online or via email because they don't know who you are, don't know who you look like, how you act etc. So they can't exactly judge you on things like that, that some people find are important”
**Accessibility**		Strong	
	Convenience and flexibility	Strong	“I can come on at any time when I feel I need to, rather than wait for the appointment with another counsellor face to face”
	Faster access to counseling	Strong	“Because I become in need of support very suddenly often when I am alone, so this means I can access support immediately”
	Affordability	Strong	“I am very young and have no way of getting to or paying for face-to-face counseling”
	Counseling in areas of low service Density	Weak	“Where I live, there isn't any services such as a therapist”
	Source one’s own counseling support	Weak	“I was restricted in a way meaning that I wasn’t able to be taken somewhere to see a councillor [sic] so this was the only way I was able to access help”
**Expectations**		Strong	
	Help with low complexity issue	Moderate	—^a^
	More helpful than other services	Moderate	—
	Comparable to other services	Moderate	“I think that online counseling is just as helpful compared to face to face counseling and that’s important to me because usually during the week, because of school, I don’t have time be taken to see a counsellor face to face”.
	Help with high complexity issues	Moderate	—
	Self-responsibility or realistic expectations	Weak	“It can be helpful to talk to people but sometimes, it's only up to yourself to fix yourself. Nobody can help you, and although it can be a bit depressing and overwhelming thinking about that, it's true”

^a^Not available.

### Selection Factors

Participants confirmed three themes related to their motivations for selecting TBOC for support: *safety*, *accessibility*, and *expectations*. A fourth theme, *avoidance motivation*, emerged from participant qualitative data. An overview of themes and subthemes related to the *selection factors* domain can be found in Table 2.

#### Safety

A majority of participants (94/100) quantitatively confirmed that the perceived safety of TBOC services was a motivator for its selection over conventional counseling services. The importance of this theme was also qualitatively described by 74% (52/70) of participants. Five subthemes were identified within the *safety* theme.

The view that TBOC felt safer due to *increased privacy* resulting from using text-based communication was quantitatively endorsed by 82% (82/100) of participants and qualitatively described by 26% (18/70) of participants. Participant responses were similar. The key aspect described was the participant’s desire to conceal details of their personal lives from those in their immediate environment, mostly parents.

The view that TBOC facilitated *honesty or communication of sensitive issues* with online counsellors due to its perceived safety was quantitatively endorsed by 82% (82/100) of participants and qualitatively described by 11% (8/70) of participants. Participant responses were similar. The key aspect described was the ability to discuss sensitive issues they might not have otherwise disclosed due to perceived *privacy*, *reduced emotional intensity of conversation,* and concealment of c*ounsellor reaction*.

Having increased *anonymity* from online counsellors due to the concealment of one’s personal identity and location that increased one’s sense of safety was quantitatively endorsed by 67% (67/100) of participants and qualitatively described by 22% (16/70) of participants. Participant responses were variable. Key aspects that emerged were about feeling safer, more comfortable, and in control during TBOC sessions and evading identification when discussing risk-related situations that the online counsellor may have a responsibility to report to authorities.

The perception that TBOC *reduced the emotional intensity of conversation* with online counsellors was quantitatively endorsed by 72% (72/100) of participants and qualitatively described by 4% (3/70) of participants. Participant responses were similar. The key aspect described was the benefit of feeling less overwhelmed when using text rather than verbal communication.

The perception that TBOC provided *increased control and autonomy* over various aspects of the counseling process was quantitatively endorsed by 56% (56/100) of participants and qualitatively described by 44% (31/70) of participants. Participant responses were variable. Key aspects that emerged were about *control* over the counseling process and *counsellor reactions* to increase one’s sense of safety in sessions and *autonomy* in accessing a mental health service, especially where parental gatekeeping was a barrier.

#### Avoidance Motivation

A parallel theme that emerged alongside the perceived *safety* of TBOC was participants’ underlying *avoidance motivation* for desiring the aforementioned benefits in the first place. This theme was qualitatively described by 64% (45/7) of participants. Four subthemes were identified within the *avoidance motivation* theme.

Selecting TBOC to avoid *being overheard* or *seen attending a service* was qualitatively described by 30% (21/70) of participants. Participant responses were similar. The key aspect described was the fear of being discovered while accessing a counseling service due to its social consequences, mostly from one’s parents.

Selecting TBOC to avoid verbal *social interaction* with a counsellor was qualitatively described by 21% (15/70) of participants. Participant responses were similar. The key aspect described was the motivation to avoid discomfort and fear associated with verbal communication.

Selecting TBOC to *minimize difficult emotions* felt during the process of counseling was qualitatively described by 17% (12/70) of participants. Participant responses were similar. The key aspect described was about text-based communication feeling less emotionally intense than verbal communication, which reduced the risk of becoming overwhelmed in a session when discussing upsetting content.

Selecting TBOC to minimize a counsellor’s potentially threatening reaction, such as judgment, in response to personal disclosures was qualitatively described by 15% (11/70) of participants. Participant responses were variable. Key aspects that emerged were about wanting to conceal judgmental reactions from the online counsellor and to circumvent other challenging reactions from the online counsellor (eg, misgendering).

#### Accessibility

A majority of participants (74/100) quantitatively confirmed that the *accessibility* of TBOC services motivated its selection over conventional counseling services. The importance of this theme was also qualitatively described by 60% (42/70) of participants. Six subthemes were identified within the *accessibility* theme.

The *convenience* of accessing TBOC was quantitatively endorsed by 66% (66/100) of participants. The *flexibility* in accessing TBOC was quantitatively endorsed by 57% (57/100) of participants and qualitatively described by 28% (20/70) of participants. Both subthemes were determined to be thematically interrelated by analysts because of construct similarity and coding of the subthemes together the majority of the time. Participant responses were variable. Key aspects that emerged were the convenience of access using one’s own device and the flexibility of access from any location or at any time of day.

The *faster access to counseling* possible with TBOC services was quantitatively endorsed by 60% (60/100) of participants and qualitatively described by 17% (12/70) of participants (17%). Participant responses were similar. The key aspect described was the benefit of being able to access support at the moment of distress or crisis instead of having to wait for a face-to-face counseling appointment.

The *affordability* of TBOC was quantitatively endorsed by 51% (51/100) of participants and qualitatively described by 17% (12/70) of participants. Participant responses were similar. The key aspect described the benefit of free services due to financial barriers young people experience to accessing paid counseling services.

The manner in which TBOC improved access to *counseling in areas of low service density* was quantitatively endorsed by 20% (20/100) of participants and qualitatively described by 4% (3/70) of participants. Participant responses were variable. Key aspects that emerged were the scarcity of counseling services where participants live and the long distance to the nearest counseling service.

The ability to *source one’s own counseling support* was qualitatively described by 11% (8/70) of participants. Participant responses were variable. Key aspects were about being able to manage one’s own mental health care and overcoming (mostly parental) gatekeeper-related obstacles to mental health care.

#### Expectations of counseling

A majority of participants (53/100) confirmed *expectations about counseling* as a motivator for using TBOC services. The importance of this theme was also qualitatively described by 20% (14/70) of participants. Five subthemes were identified within the *expectations* theme.

Expectations that TBOC would *help with low complexity issues* (evaluated by a proxy question inquiring about “short-term issues”) was quantitatively endorsed by 41% (41/100) of participants.

Expectations that TBOC was *more helpful than other services* used previously was quantitatively endorsed by 37% (37/100) of participants.

Expectations that TBOC services would be *comparable to other services* in effectiveness was quantitatively endorsed by 36% (36/100) of participants and qualitatively described by 4% (3/70) of participants. Participant responses were similar. The key aspect described was the belief that all modalities of counseling were comparably effective.

Expectations that TBOC would *help with high complexity issues* (evaluated by a proxy question inquiring about “long-term issues”) was quantitatively endorsed by 31% (31/100) of participants.

*Having self-responsibility* over one’s process of change in counseling or *realistic expectations* of what TBOC was able to offer was qualitatively described by 17% (12/70) of participants. Participant responses were variable. Key aspects that emerged were having no *expectation* that the online counsellor could help them and believing it was their own responsibility to create change in their lives.

### Factors Perceived to Increase Effectiveness

Participants confirmed three themes related to perceptions about factors that increased the effectiveness of TBOC: *general therapeutic benefits*, *persisting with counseling to increase benefit*, and *efficacious modality and service factors*. An overview of themes and subthemes related to the *factors perceived to increase effectiveness* domain can be found in Table 3.

**Table 3:** Overview of themes and subthemes related to the *factors perceived to increase effectiveness* domain that were confirmed and identified in the study.

Themes and subthemes		Strength	Examples
**General therapeutic benefits**		Strong	
	Feeling heard and understood	Strong	“The feeling of someone listening and understanding you helps you feel like you're not invisible”
	Catharsis or debriefing	Strong	“To finally be able to talk about something I haven't been to talk about with anyone else and have even kept it from the professionals I see is very relieving”
	Feeling normalized and validated	Strong	“I felt very safe and calm when I was talking to them because they made me feel important and like I was worth listening to no matter how big or small my problem was”
	Rapport and feeling supported	Moderate	“It was really good to feel like someone was interested in what I was saying and cared about helping me”
	Outsider perspective and support	Weak	“It felt good to speak to someone who doesn't actually know you as they can't judge you on what you speak about. They also offer and insight that I might not have thought of”.
	Problem clarification	Weak	“by talking to [online counsellors] I could understand my haywire emotions”.
Persisting with counseling to increase benefit		Strong	“After a while I guess it does feel like the problems aren't solved but they do get better with more and more chats.”
**Positive modality and service factors**		Moderate	
	Facilitating expression and thought organization	Weak	“Because I get anxious talking to someone face to face and usually forget everything that I am feeling or that I am going to say but when I can type it out and think about what I’m saying”

#### General Therapeutic Benefits

A majority of participants (79/100) quantitatively confirmed that *general therapeutic benefits* were related to the effectiveness of the counseling process. The importance of this theme was also qualitatively described by 65% (46/70) of participants. Seven subthemes were identified within the *general therapeutic factors* theme.

*Feeling heard* (ie, “Being listened to”) was quantitatively endorsed by 77% (77/100) of participants. *Feeling understood* was quantitatively endorsed by 72% (72/100). Due to the overlap between participant’s qualitative descriptions of feeling “listened to” and “understood,” both were collapsed into a single subtheme. The effectiveness of both *feeling heard and understood* by their online counsellor was qualitatively described by 24% (17/70) of participants. Participant responses were variable. Key aspects that emerged were facilitating engagement with the counsellor, helping them feel comfortable, helping them feel affirmed, reducing their sense of invisibility, and reducing their sense of being alone in the session.

The effectiveness of *catharsis or debriefing* was quantitatively endorsed by 64% (64/100) of participants and qualitatively described by 21% (15/70) of participants. Participant responses were similar. The key aspect described the benefit of debriefing one’s personal situation with an online counsellor or feeling relief from doing so.

The effectiveness of *feeling normalized and validated* was quantitatively endorsed by 61% (61/100) of participants and qualitatively described by 8% (6/70) of participants. Participant responses were variable. Key aspects that emerged were about feeling accepted by the online counsellor, less alone with problems they were facing, and that their experiences were taken seriously.

The effectiveness of *rapport and feeling supported* was qualitatively described by 34% (24/70) of participants. Participant responses were similar. The key aspect described the benefit of feeling like the online counsellor cared about them and showed interest in helping them.

The effectiveness of *outsider perspective and support* was qualitatively described by 15% (11/70) of participants. Participant responses were similar. The key aspect described was the benefit of talking to a person outside of one’s social network with an objective perspective about their presenting problem.

Perceiving the process of *problem clarification* via TBOC to contribute to its effectiveness was qualitatively described by 11% (8/70) of participants. Participant responses were similar. The key aspect described was the benefit of being assisted to better understand their presenting problems.

#### Persisting With Counseling to Increase Benefit

A majority of participants (56/100) quantitatively confirmed benefits associated with accessing TBOC over multiple sessions. The importance of this theme was also qualitatively described by 47.1% (33/70) of participants. Participant responses were variable. Key aspects that emerged were about persisting with contact due to benefits of *general therapeutic factors*, short-term benefits such as distraction and crisis-based support, and the belief that more sessions were associated with greater improvement.

#### Positive Modality and Service Factors

Factors perceived to increase effectiveness related to TBOC as a service and modality were confirmed by 47% (33/70) of participants. Six themes were identified within the *factors perceived to increase effectiveness* domain.

Perceiving that text communication was effective in *facilitating expression and thought organization* was qualitatively described by 14% (10/70) of participants. Participant responses were variable. Key aspects that emerged were the ease in articulating one’s ideas compared to verbal communication and the better ability to organize one’s thoughts in sessions.

### Factors Perceived to Decrease Effectiveness

Participants confirmed two themes related to perceptions about what decreased the effectiveness of TBOC: *persisting with counseling despite benefit* and *negative modality and service factors*. An overview of themes and subthemes related to the *factors perceived to decrease effectiveness* domain can be found in Table 4.

**Table 4:** Overview of themes and subthemes related to the *factors perceived to decrease effectiveness* domain that were confirmed and identified in the study.

Themes and subthemes		Strength	Examples
Persisting with counseling despite benefit		Strong	“After talking to them for like the 10 time I didn't seem to feel much better than when I started talking”
**Negative modality and service factors**		Strong	
	Problem not improving or ineffective techniques	Weak	“they wouldn't give me any real advice or opinion on my topic...”
	Poor timeliness of response	Weak	“the only unhelpful thing is that often you guys are overly busy and not always able to connect with people”
	Poor conversation of counseling into postsession action	Weak	“Even though I had discussed the problem I found it hard to make an action after the session to help solve the problem”

#### Persisting With Counseling Despite Benefit

A majority of participants (53/100) quantitatively confirmed that they continued to use TBOC despite substantial progress in resolving their presenting problem. This theme was also qualitatively described by 47.1% (33/70) of participants. Key aspects that emerged were about persisting with contact due to the inability to overcome chronic presenting problems such as mental ill health, realizing the short-term benefits of counseling had failed to produce long-term outcomes, dissatisfaction with one’s *problem not improving* or *ineffective techniques* and a *poor conversation of counseling into post-session action,* and the belief that multiple sessions might be associated with greater improvement.

#### Negative Modality and Service Factors

A majority of participants (53/100) quantitatively confirmed *negative modality* and *service factors* as a theme comprised of themes related to difficulties making progress toward resolving a presenting problem. Four subthemes were identified within this theme.

Perceiving the effectiveness of TBOC to be lower due to a participant’s *problem not improving* or *ineffective techniques* being utilized was qualitatively described by 18% (13/70) of participants. Participant responses were variable. Key aspects were TBOC generally not helping them resolve their presenting problem to a satisfactory degree and the experience of initially “feeling better” followed by the observation that the effects were not sustained.

Perceiving the effectiveness of TBOC to be lower due to the *poor timeliness of response* to a participant’s session request was qualitatively described by 14% (10/70) of the participants. Participant responses were variable. Key aspects were negative feelings about having to wait for long periods to talk to an online counsellor and suggestions for service improvements to allow participants to make a more informed decision about whether to continue waiting during long queues or abandon one’s efforts (eg, queue position indicator).

Perceiving the effectiveness of TBOC to be lower due to a *poor conversation of counseling into post-session action* was qualitatively described by 10% (7/70) of participants. Participant responses were similar. The key aspect described was about not knowing or having discussed with the online counsellor about what postsession action would help them progress toward resolving their presenting problem.

## Discussion

### Principal Findings

The aims of this study were to confirm and expand on domains and themes related to the characteristics of young service users accessing TBOC services and their motivations and perceptions of TBOC effectiveness.

In terms of *user characteristics*, demographic data confirmed that female-identifying participants living in urban areas made up the majority of the sample. This is in line with the literature suggesting that women comprise the majority of western service users and are believed to access TBOC in greater numbers due to its greater safety and accessibility [40,48-51]. Diagnosis with a *mental or physical health* syndrome was also highlighted, consistent with previous research [31,38,39,48,58] and Australian prevalence estimates [61]. Interestingly, there was a higher proportion of personality, posttraumatic stress, and eating syndromes than expected in the sample. However, this was in line with the observations of online counsellors in research [62,63] and may reflect the higher patterns of distress and problem burden reported by young service users on these modalities [39,50,51]. Uniquely, a quarter of participants reported *perceived social difficulties* as underpinning their selection of TBOC services. Although young people’s preference for text-based communication is widely established in e-mental health literature, it is often attributed to cultural or technology preferences rather than anxieties about one’s social abilities [53-55]. This finding may contextualize various dimensions of TBOC use regarding user characteristics (eg, anxiety presentations), selection motivations (eg, safety and avoidance), and factors perceived to moderate effectiveness (eg, facilitating expression and thought organization). However, as some participants reported developmental (7/100) and learning syndromes (5/100), further research is required to clarify factors that influence these perceived difficulties.

In terms of *selection factors*, participants confirmed *safety* and its subthemes as the strongest motivators for accessing TBOC services, consistent with e-mental health literature [30,36,49,53]. Similarly, participants confirmed *accessibility* as a strong motivator for selecting TBOC services with subthemes that mirror research about the aspects of these services that help young service users overcome financial, gatekeeper, and transportation barriers to mental health care [9,10,64,65]. Surprisingly, the advantages TBOC offers in providing *counseling in areas of low service density* and *sourcing one’s own counseling support* were weaker themes. One possible explanation is that campaigning, resourcing, and servicing efforts in youth mental health have increased access to services such that they are not considered to be as important as they once were. *Expectations of counseling* were confirmed as another strong motivator for accessing TBOC services, in which participants described a dichotomy of subthemes about holding low-high expectations of the outcome of counseling. This too is consistent with the existing literature describing how young service users often do not know what to expect from e-mental health services or how they hold very high expectations of them [37,56]. Lastly, *avoidance motivation* emerged as a unique and strong motivator for participants in accessing TBOC services, with subthemes that paralleled and provided context for a participant’s desire for *safety* factors. Interestingly, these subthemes were reported with much weaker strength than their *safety* counterparts, possibly pointing to nuanced differences between idealized and nonnegotiable aspects of service selection. Another possibility is that this theme was underreported due to measurement limitations in comparing quantitative (*safety* factors) with qualitative (*avoidance motivation* factors) data.

In terms of *factors perceived to increase effectiveness*, *general therapeutic benefits* were confirmed as a strong theme, with subthemes indicating the importance that participants place on interpersonal and relational aspects of the counseling process. These results are novel in their specificity about the *general therapeutic benefits* young service users find most helpful when working in TBOC environments, especially *problem clarification*, which has been associated with improved outcome in at least one study [40]. They are also unsurprising in view of the link between therapeutic alliance and outcome in working with young people via e-mental health services [66] and the fact that person-centered psychotherapy is one of the most common approaches to counseling at youth-focused helplines [67]. In contrast, *efficacious modality* and *service factors* were focused on the modality *facilitating expression* and *thought organization* in discussing a participant’s presenting problems. The substantially lower strength of this theme was surprising, given its emphasis in the literature [53-55]. Nevertheless, this may reflect participants’ perceptions of what works for their unique presenting problem, which may have resulted in a diversity of suggestions with little overlap. *Persisting with counseling to increase benefit* was also confirmed by participants as a means to increase the effectiveness of support, in line with research indicating that multiple sessions of TBOC are superior to single sessions [50]. Findings suggest that a positive feedback loop of *factors perceived to increase effectiveness* may contribute to young service user’s persisting behavior. Nevertheless, further research is required to determine whether other factors may also encourage young service users to attend multiple TBOC sessions (eg, psychoeducation and help-seeking beliefs).

Finally*, factors perceived to decrease effectiveness* was confirmed as a strong domain with two themes. *Persisting with counseling despite benefit* was confirmed to be a strong theme, for which participants described experiencing various *factors perceived to decrease effectiveness* during TBOC sessions. This is an interesting finding that raises important questions about the reasons why young service users might persist using TBOC services when they are perceived to be of insufficient benefit. One possibility is that young service users may experience challenges in identifying or taking action about their declining treatment progress with online counsellors, resulting in repeated session attendance until the disadvantages of unproductive sessions become more apparent. This would be a valuable area of future research, given the increased probability of dropout likely from continued service ineffectiveness, which has been described as a contributing factor to the high dropout rates observed in e-mental health effectiveness research [68,69]. *Negative modality* and *service factors* produced three themes. Herein, *poor timeliness of response* appears to be a common complaint made by young service users about TBOC services [49], which may point to the unmet need resulting from low staffing relative to high demand. *Problems not improving* or *ineffective techniques* has also been inferred as an issue in TBOC process literature from the perspective of online counsellors in relation to modality-specific issues, such as heightened client anonymity, limitations in text-only communication, and the slow pace of typed sessions [62,63,70]. Finally, the *poor conversion of counseling into postsession action* was a unique finding in view of the fact that action planning processes are commonly utilized techniques by online counsellors [40]. Our findings may suggest that other factors complicate action planning for online counsellors, such as the aforementioned modality-specific limitations combined with a disproportionate amount of time spent building rapport and clarifying problems [40,62].

### Implications for Online Counsellors

Our findings have several implications for youth-focused TBOC service model development and online counsellor training. First, awareness of common user characteristics and motivations for accessing TBOC services may help online counsellors better understand young service users’ presenting problems and determine the best clinical course during sessions. Additionally, awareness of the *avoidance motivations* underlying a young service users’ desire for *safety*, *accessibility*, and *expectations of counseling* may provide online counsellors with important insight that helps guide their selection of specific counseling interventions to use on TBOC modalities. For example, there may be an iatrogenic effect for young service users selecting TBOC services to compensate for perceived social deficits and an important role for employing exposure interventions that aim to collaboratively transition to verbal communication modalities. Finally, an awareness of *factors that increase and decrease effectiveness* of TBOC services has important implications for an online counsellor’s therapeutic process. Our findings suggest that young service users may benefit from a combined *humanistic and solution*-focused counseling approach that balances supportive and problem-solving interventions as well as the incorporation of progress-monitoring practices to detect early markers of treatment ineffectiveness that lead to dropout. Additionally, busy TBOC service providers may benefit from software that provides feedback to young service users about their expected waiting time before speaking to an online counsellor, to help users make an informed decision about waiting in long queues or abandoning their efforts.

### Limitations

There are three main limitations to this study that may affect the generality of the findings. First, although many findings were consistent with those in the existing literature, the sample was small and consisted of self-selected female participants above the age of 15 years, whose perceptions of TBOC may not align with the concerns of other genders or age cohorts. Further research is required to confirm the validity and generality of the reported domains and themes. The absence of comparator modalities (eg, SMS, forums, and social media groups) and relatively lower proportion of email counseling users also limits the conclusions that can be drawn about the generality of TBOC domains and themes. Having nontext–based e-mental health comparators would add further validity as well as a form of manipulation check to our findings. Finally, quantitatively measured domains and themes may have been overrepresented due to the lower effort required to respond to them as compared to drafting a qualitative response. Therefore, further research to confirm the validity of qualitative-only themes (eg, *factors perceived to increase and decrease effectiveness*) would be important.

### Conclusions

This study examined the user characteristics and motivations of young service users accessing TBOC services and their perceptions about factors influencing its effectiveness. The study found that many participants selected TBOC services to increase their sense of safety in response to negative perceptions of their social skills and the online counsellor’s response to their presenting problem. By using such services, they also sought to improve their accessibility to mental health services that better met their expectations. Factors that increased effectiveness of TBOC were the counsellor’s interpersonal skills and using text communication. Factors that reduced its effectiveness were related to poor timeliness in response to their service requests, experiencing no change in their presenting problem, and not knowing what postcounseling action to take. Online counsellors need to be aware of these TBOC factors in order to best assess, educate, and respond to the needs of their clientele and provide them with the best therapeutic experience possible.

## Appendix

Multimedia Appendix 1 Full pilot study survey.

## Abbreviations

e-mental: electronic mental
SMS: short message service
TBOC: text-based online counseling
