# Supplementary material for: Exploring Young People’s Perceptions of the Effectiveness of Text-Based Online Counseling: Mixed Methods Pilot Study
Source: JMIR Ment Health. 2019 Jul 3;6(7):e13152. doi: 10.2196/13152 (PMC6636236; doi:10.2196/13152)
Supplement: Multimedia Appendix 1 [file mental_v6i7e13152_app1.pdf]

Multimedia Appendix 1:
Complete Study Survey

What is your age?
Age: ________years _____months

What is your gender?
- ☐ Male
- ☐ Female
- ☐ Trans or Gender Fluid
- ☐ Intersexed
- ☐ Other

What is your ethnicity or cultural background?
- ☐ Caucasian or European descent
- ☐ Hispanic or Latino
- ☐ Black or African American
- ☐ Asian
- ☐ Middle Eastern
- ☐ Aboriginal or Torres Straight Islander
- ☐ Other

Do you follow any religion?
- ☐ None
- ☐ Christianity
- ☐ Islam
- ☐ Judaism
- ☐ Buddhism
- ☐ Hindu
- ☐ Shinto
- ☐ Taoism
- ☐ Other

What is the highest degree or level of schooling you have completed or are currently completing?
- ☐ No schooling completed
- ☐ Kindergarten to 7th grade
- ☐ Some high school, no diploma
- ☐ High school graduate or TAFE equivalent
- ☐ Some university credit, no degree
- ☐ Trade/technical/vocational training
- ☐ Bachelor's degree
- ☐ Postgraduate degree

What is your relationship status?
- ☐ Single, never married
- ☐ In a dating relationship
- ☐ Married or de facto partner
- ☐ Widowed

- Divorced
- Separated

Who lives at home with you? (tick all that apply)

- Both parents
- Single parent
- Sibling/s
- Grandparent/s
- Uncle/s or Aunt/s
- Cousin/s
- Boyfriend or Girlfriend
- Friend/s and/or friend's parents

What is the highest level of schooling that your parents/guardians/caretakers have completed? (If unknown, how much education do you think they may have completed?)

Person 1:

- No schooling completed
- Kindergarten to 8th grade
- Some high school, no diploma
- High school graduate or TAFE equivalent
- Some college credit, no degree
- Trade/technical/vocational training
- Bachelor’s degree
- Master’s degree
- Doctorate degree

Person 2:

- No schooling completed
- Kindergarten to 8th grade
- Some high school, no diploma
- High school graduate or TAFE equivalent
- Some college credit, no degree
- Trade/technical/vocational training
- Bachelor’s degree
- Master’s degree
- Doctorate degree

What is your current job?

- Employed
- Unemployed
- Stay-at-home caretaker
- Student
- Military
- Unable to work

Do you currently have, or have you ever, been diagnosed with a chronic physical health illness?

- ☐ Yes
- ☐ No

***If yes, which chronic physical health illnesses have you been diagnosed with? (Tick all that apply)**

- ☐ Heart or Cardiovascular Disease (e.g. blood pressure, irregular heartbeat,
- ☐ Thyroid disease (e.g. inactive thyroid gland, hyperthyroidism)
- ☐ Cancer
- ☐ Chronic Kidney Disease
- ☐ Diabetes
- ☐ Respiratory Disease (e.g. Asthma)
- ☐ Muscular or Bone-related Diseases (e.g. osteoporosis, arthritis)
- ☐ Obesity
- ☐ Substance Misuse or Abuse (e.g. tobacco, alcohol, recreational drugs)
- ☐ Food or Environmental allergies
- ☐ Autoimmune Disease (e.g. Multiple Sclerosis, Crohn's Disease, Lupus)
- ☐ Neurological Disease (e.g. Epilepsy, Parkinson's disease, Cerebral Palsy, Brain Injury)
- ☐ Blood-related Disease (e.g. HIV)
- ☐ Genetic Disease (e.g. Down Syndrome, Huntington's Disease, Muscular Dystrophy,)
- ☐ Vision-related Disease (e.g. Glaucoma, partial blindness)
- ☐ Other

Do you currently have, or have you ever, been diagnosed with a mental health syndrome?

- ☐ Yes
- ☐ No

***If yes, which mental health syndromes have you been diagnosed with? (Tick all that apply)**

- ☐ Mood Syndrome (e.g. depression, bipolar disorder)
- ☐ Anxiety Syndrome (e.g. generalised anxiety, social anxiety disorder, obsessive compulsive disorder)
- ☐ Schizoprenia
- ☐ Post-Traumatic Stress Disorder
- ☐ Personality Syndrome (e.g. Borderline Personality Disorder, Antisocial Personality Disorder)
- ☐ Substance Misuse or Abuse Syndrome
- ☐ Eating Syndrome (e.g. Anorexia Nervosa, Bulimia Nervosa)
- ☐ Childhood (e.g. ADHD, Asperser's Syndrome, Oppositional Defiant Disorder, Separation Anxiety)
- ☐ Learning Disability
- ☐ Other

Thank you for helping Kids Helpline with this research study. In this questionnaire we'd like to hear about your experiences using email and webchat counselling at Kids Helpline or any similar service similar that uses text-based counselling. We're especially interested in hearing about the times that you haven't found these services helpful in overcoming a problem you talked to a counsellor about. Your answers will help us better understand how to make Kids Helpline's online service more helpful for people in the future.

1. Have you ever used email or webchat counselling?

☐ Yes ☐ No*

**Go to end of survey*

2. How many times would you guess that you've used email or webchat counselling before?

☐ 0-1* ☐ 2-4 ☐ 4-10 ☐ 10+

**Go to end of survey*

3. We talk to a lot of young people about the reasons they decide to use email and webchat counselling *instead of face-to-face counselling*. Here are some of the reasons we hear about often:

3a. ***Accommodation reasons:*** Many young people say that they find email and webchat counselling much easier to fit around their lives, compared to face-to-face services. Some people like the:

- **Low Cost** (i.e. free or minimal cost of contacting);
- How easy online services are to **Access** from home or other places, and that;
- Fact there is a service they can access since there are **No Services Available** near them, even if they found a way to travel to one by public transport;
- You can be **Flexible** with the day and time you want to talk to a counsellor.

Please select the ***Accommodation reasons*** that made you decide to use email and webchat counselling (select as many as you like):

☐ None of these contributed to my decision*
☐ Low Cost
☐ Access
☐ No Services Available
☐ Flexibility

**Go to next question*

Can you tell us more about why the ***Accommodation reasons*** you selected (if any) were important to you when you first started using email/webchat counselling?

Are there any ***Accommodation reasons*** we haven't listed that were reasons were important to your decision to use email/webchat counselling services?

3b. ***Safety reasons*:** Many young people say that they find email and webchat counselling much safer to use than face-to-face services. Some people like how online calls:

- Feel more **Private**;
- They can be more **Anonymous**;
- They can receive a **Faster Response** than having to wait for an appointment;
- They feel more **Comfortable Talking Online** because they don't think they have good social skills;
- They feel more in **Control** of the conversation online and when they can leave;
- They feel their emotions or symptoms are **Less Overwhelming** when talking about them online;
- They find online services **More Helpful** than other services they have used.

Please select the ***Safety reasons*** that made you decide to use email and webchat counselling (select as many as you like):

☐ None of these contributed to my decision*
☐ Privacy
☐ Anonymity
☐ Faster Response
☐ Comfort talking Online
☐ Control
☐ Emotions or Symptoms less Overwhelming
☐ More Helpful

**Go to next question*

Can you tell us more about why the ***Safety reasons*** you selected (if any) were important to you when you first started using email/webchat counselling?

Are there any ***Safety reasons*** we haven't listed that were reasons were important to your decision to use email/webchat counselling services?

3c. ***Effectiveness reasons.*** Many young people say that they find email and web counselling more effective than face-to-face services. Some people use email and web counselling because:

- Email and webchat counselling helped them **Overcome a Short-term Problem** they were having (e.g. making a decision, understanding a problem better, getting a referral, getting through a hard day);
- Email and webchat counselling hasn't help them **Overcome a Long-Term Problem** they were having (e.g. completely overcoming a mental health problem);
- They think email and webchat counselling are **Probably as Helpful** as any other type of counselling.

Please select the ***Effectiveness reasons*** that made you decide to use email and webchat counselling (select as many as you like):

☐ None of these contributed to my decision*
☐ Overcome a Short-term Problem
☐ Overcome a Long-term Problem
☐ Probably as Helpful as other counselling

**Go to next question*

Can you tell us more about why the ***Effectiveness reasons*** you selected (if any) were important to you when you first started using email/webchat counselling?

Are there any ***Effectiveness reasons*** we haven't listed that were reasons were important to your decision to use email/webchat counselling services?

4. There are many reasons that young people tell us that they like using email and web counselling. Some of the reasons are that talking to a counsellor helps them feel **heard**, **validated**, **normalised**, and **relieved after talking.** Have you ever had an experience like this before when using email or webchat counselling?

☐ Yes* ☐ No

*Can you tell us more about what your experience of this was like?

5. There are many reasons young people tell us that they like using email and web counselling. Some of the reasons are that talking to a counsellor online makes them feel **more uncomfortable** and they have to **worry less** about:

- How private and anonymous they are talking to a counsellor face-to-face;
- Feeling emotionally overwhelmed about talking to a counsellor face-to-face;
- Feeling like they might be judged if they talk to a counsellor face-to-face;
- Feeling like their symptoms might get worse if they talk to a counsellor face-to-face;
- Not wanting to talk to their usual workers who they don't always get along with;
- Feeling like they don't have the social skills to talk to someone face-to-face;
- Feeling more in control of the conversation and when they can leave, or;
- Whether they can wait for the next appointment with another support service.

Have you ever had an experience like this before when using email or webchat counselling?

☐ Yes* ☐ No

*Can you tell us more about what your experience of this was like?

6. Some young people use email and web counselling find that ***while it is helpful to talk to someone at first***, they notice over time that talking ***doesn't help them overcome the problem they need help with***. Have you ever had an experience like this before when using email or webchat counselling?

☐ Yes* ☐ No

*Can you tell us more about what your experience of this was like?

7. Some young people who use email and web counselling find that ***while talking doesn't help them overcome the problem they need help with***, they ***like talking to a counsellor anyway*** and so keep using the service anyway. Have you ever had an experience like this before when using email or webchat counselling?

☐ Yes* ☐ No

*Can you tell us more about what your experience of this was like?

8. Are there any other aspects of email and webchat counselling that you've found helpful or unhelpful that would be important for us to know about?

Thank you for your time and assistance today.
